# Pulsed-Field Ablation in Adult Congenital Heart Disease: Promise, Prudence, and the Path Forward

**DOI:** 10.1016/j.jaccas.2025.106742

**Published:** 2026-01-21

**Authors:** Konstantinos N. Aronis

**Affiliations:** Section of Cardiac Electrophysiology, Department of Medicine, Johns Hopkins Hospital, Baltimore, Maryland, USA

**Keywords:** ablation, atrial fibrillation, congenital heart defect, ventricular tachycardia

**Figure:**
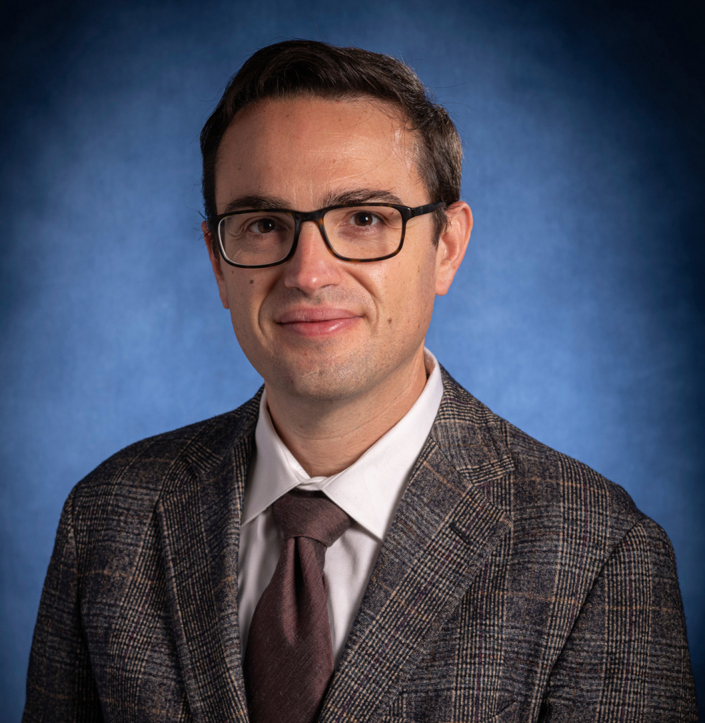

Adult congenital heart disease (ACHD) patients with arrhythmias face unique challenges stemming from their underlying anatomy, surgical repair, and natural progression of their disease. With improvements of surgical techniques, the number of ACHD patients who reach adulthood is increasing, and currently there are more adults with congenital heart disease than children.^1^ Arrhythmias are a leading cause of morbidity and mortality in ACHD patients.^1^ Catheter ablation approaches using radiofrequency energy or cryoablation have been successfully used, but they can be limited by complex anatomy, extensive surgical scarring, and heightened risks of collateral injury in these patients.^2^

The introduction of pulsed-field ablation (PFA) has led to a paradigm shift in electrophysiology, as it delivers tissue-selective nonthermal ablation. This may address many of the inherent challenges associated with ablation in ACHD patients. In this issue of *JACC: Case Reports*, the report by Iqbal et al^3^ demonstrates the successful use of the Sphere-9 PFA catheter in complex ACHD patients and provides a valuable real-world demonstration of this technology's potential. Importantly, the Sphere-9 system's dual-energy capability—enabling both pulsed-field and radiofrequency ablation through a single catheter—offers unique flexibility in ACHD patients. This integrated map-and-ablate platform allows operators to tailor energy delivery to specific anatomic constraints, switching to radiofrequency when PFA may pose a heightened risk, such as near the coronary arteries or metallic implants. However, as we embrace this innovation, it is crucial to examine both the promise and limitations of PFA in this specialized patient population.

PFA has several advantages over thermal ablation energy sources. First, PFA is tissue selective.^4^ It creates irreversible electroporation through high-voltage electrical pulses that preferentially affect cardiac myocytes while sparing adjacent structures.^5^^,^^6^ This selectivity is particularly relevant in ACHD patients, where critical structures such as the esophagus and phrenic nerve may be near ablation targets owing to altered anatomy or surgical reconstruction. Early clinical experience suggests that PFA's nonthermal mechanism significantly reduces the risk of thermal collateral injuries that have historically complicated radiofrequency ablation in ACHD patients.^5^ Second, the preservation of extracellular matrix and endothelial function may also contribute to more favorable healing characteristics, potentially reducing the risk of pulmonary vein stenosis—a particular concern in patients with already compromised hemodynamics. Third, PFA has the potential to significantly reduce the procedure time. Real-world registries consistently demonstrate high first-pass pulmonary vein isolation rates and shortened left atrial dwell times compared with conventional ablation.^7^^,^^8^ This procedural efficiency is especially valuable in ACHD patients, who often require longer procedures owing to their complex anatomy, difficult vascular access, and the need for extensive mapping. Last, the potential of PFA to achieve transmural lesions in fibrotic tissue is critical for ablation in ACHD patients. In preclinical studies, PFA can effectively create transmural lesions over chronic radiofrequency scars and homogenize previously ablated tissue.^9^ Many ACHD patients have extensive postsurgical scarring and may require repeat procedures. The ability to ablate areas with existing fibrosis could significantly improve long-term outcomes. The accompanying case report's demonstration of successful PFA in a complex ACHD anatomy supports this potential advantage. Notably, this series extends beyond atrial arrhythmias to include ventricular tachycardia ablation in 3 ACHD patients, an application with even more limited published evidence. The successful acute outcomes in these ventricular tachycardia cases suggest that the advantages of PFA may also apply to ventricular substrate ablation in congenital hearts, although long-term efficacy data are needed.

While PFA's safety profile appears favorable in the general population, ACHD patients present unique anatomic and physiologic challenges that require careful consideration. The complex anatomy of repaired hearts, presence of surgical baffles and conduits, and altered electrical conduction patterns may influence procedural risk in ways that are not yet fully understood for PFA technology.^10^, ^11^, ^12^ Effective delivery of PFA energy requires stable catheter contact and appropriate tissue proximity, which may be challenging to achieve in these altered anatomies. The presence of metallic surgical materials raises theoretical concerns about electrical field distribution and potential device interactions that have not been systematically studied.^11^ Vascular access remains a significant challenge in many ACHD patients, with some requiring transseptal puncture through baffles or alternative access routes. The safety of PFA delivery via various access approaches in proximity to surgical materials requires further investigation. PFA carries the risk of affecting nearby coronary arteries, especially in patients with anomalous coronary anatomy or surgical coronary reimplantation. The long-term effects of PFA on valvular function, particularly in patients with prosthetic or repaired valves, remain unclear. The accompanying case series outlines practical mitigation strategies for these concerns, including preprocedural imaging to define coronary and phrenic nerve proximity, intraprocedural switching to radiofrequency energy when anatomic risk is identified, and the use of intracardiac echocardiography to guide catheter positioning. These pragmatic approaches merit validation in larger cohorts.

The enthusiasm for PFA in ACHD must be tempered by recognition of significant evidence gaps that limit our ability to make definitive recommendations about its use in this population.^13^ The current literature consists primarily of small case series, registry subgroup analyses, and extrapolations from general population studies.^14^ The ACHD PFA experience remains limited, with most published series including fewer than 20 patients and representing highly selected cases from expert centers. This selection bias likely underestimates both the complexity of typical ACHD cases and the potential complications that might occur with broader adoption. Perhaps most critically, there are no randomized controlled trials or large comparative studies examining PFA versus conventional ablation, specifically in ACHD patients. The assumption that PFA's advantages in structurally normal hearts translate to ACHD patients requires further validation. Furthermore, the durability of PFA lesions in patients with ACHD remains unknown. The complex substrates of postsurgical scars, fibrosis, and altered tissue properties may influence lesion formation and persistence in ways that differ from those of typical atrial fibrillation substrates. Long-term follow-up data examining arrhythmia-free survival, quality of life, and hard clinical endpoints such as stroke and mortality are essential, but they are currently lacking. Finally, the ACHD population encompasses an enormous spectrum of anatomic complexities, from simple repaired lesions to complex single-ventricle physiology. Published ablation studies have historically over-represented simpler lesions while under-representing the most complex cases that might benefit the most from the PFA's potential advantages. This limits the generalizability of the current findings to the broader ACHD population. Table 1 summarizes the critical steps that need to be taken as we consider the integration of PFA into ACHD practice.

**Table 1:** Future Directives for PFA in ACHD

Domain	Future Directive	Practical Focus	Target Population/Setting	Desired Outcomes
Clinical trials	Conduct multicenter, prospective ACHD PFA trials	Evaluate PFA in prespecified ACHD subgroups, with standardized safety endpoints and ≥2-y follow-up	ACHD with AF or other atrial arrhythmias across anatomic complexity spectrum	Arrhythmia-free survival, QoL, stroke, mortality, complication rates
Anatomic safety	Systematically map PFA safety near critical structures	Use intraprocedural imaging and, when appropriate, intracardiac echocardiography and invasive coronary assessment to define safe energy delivery zones	ACHD with surgical baffles, conduits, coronary proximity, valve prostheses/repairs	Anatomy-specific risk profiles; procedural safety guidelines
Redo ablation substrate	Study PFA in prior RF/ablation scar	Evaluate efficacy and safety of PFA for arrhythmias arising from chronic postsurgical or RF scars	ACHD with prior ablations, dense atrial scarring, complex VT/AT substrates	Acute success, durable lesions, reduced need for repeat procedures
Real-world registries	Establish ACHD-focused PFA registries	Capture procedural techniques, access strategies, imaging use, complications, and longitudinal outcomes	High-volume ACHD/EP centers using PFA	Best-practice patterns, patient selection criteria, benchmark outcomes
Center designation	Concentrate PFA in specialized ACHD/EP centers	Require combined ACHD and advanced EP expertise, multimodality imaging, complex access, and complication management capability	ACHD patients considered for PFA	Improved safety, consistency, and generalizability of outcomes
Training and learning curve	Formalize PFA training pathways in ACHD	Structured mentorship, proctoring, and graded increase in anatomic/procedural complexity	Operators and centers early in ACHD PFA adoption	Reduced complications, standardized competence milestones
Technology development	Design ACHD-optimized PFA catheters/systems	Catheters adaptable to distorted atria, baffles, conduits, and single-ventricle anatomy	ACHD with complex geometry (eg, Fontan, Mustard/Senning, cc-TGA)	Improved lesion delivery, access to difficult targets
Imaging integration	Refine imaging guidance for PFA	Integrate 3D echocardiography, CT/MRI fusion, and intracardiac imaging into PFA workflows in altered anatomy	ACHD with prior surgery, patches, systemic venous rerouting	Safer energy delivery, better lesion placement, fewer complications
Access strategies	Standardize baffle puncture and alternative access	Develop evidence-based protocols and tools for baffle puncture, transhepatic, surgical-hybrid, and alternative venous access	ACHD with atrial baffles, occluded veins, complex systemic–pulmonary connections	Higher procedural success, fewer access-related complications
Mapping integration	Combine high-density mapping with PFA	Protocolize use of ultra–high-density activation/voltage mapping to guide PFA lesion sets	ACHD with heterogeneous scars and non-PV substrates	More tailored lesion sets, higher durable arrhythmia control
Outcome standardization	Establish uniform definitions and metrics	Standardize procedural success definitions, safety endpoints, QoL measures, and benchmark outcomes across studies/centers	All ACHD PFA studies and registries	Consistent reporting, reproducible comparisons, quality improvement tracking

This table summarizes key priorities for advancing PFA in the ACHD population. Eleven domains are identified spanning clinical research, safety evaluation, technology development, training, and outcome standardization. For each domain, the table outlines the specific directive, practical implementation focus, target population or clinical setting, and anticipated outcomes. These recommendations aim to guide the safe and effective integration of PFA into ACHD electrophysiology practice.

3D = 3-dimensional; ACHD = adult congenital heart disease; AT = atrial tachycardia; cc-TGA = congenitally corrected transposition of the great arteries; CT = computed tomography; EP = electrophysiology; MRI = magnetic resonance imaging; PFA = pulsed-field ablation; PV = pulmonary vein; QoL = quality of life; RF = radiofrequency; VT = ventricular tachycardia.

The case series by Iqbal et al^1^ demonstrates the feasibility of dual-energy ablation using the Sphere-9 catheter across a spectrum of complex ACHD patients, including both atrial and ventricular arrhythmias. Their experience represents an important early milestone in the evolution of arrhythmia management for this challenging population. However, enthusiasm must be balanced against significant knowledge gaps and the unique anatomical risks inherent to congenital heart disease. Moving forward, the electrophysiology community must prioritize rigorous clinical research, maintain a focus on specialized center care, and continue technical innovation to realize the full potential of PFA in ACHD patients.

## Funding Support and Author Disclosures

Dr Aronis has received honoraria from Johnson and Johnson MedTech (for presentation at educational conferences), Future Cardia, Atricure, and Impulse Dynamics outside the submitted work.
